# Perioperative Methadone in Orthopedic Surgery: A Scoping Review

**DOI:** 10.3390/healthcare13192431

**Published:** 2025-09-25

**Authors:** Albert F. Yang, Emily Lee, Mahsa Babaei, Paul Lee

**Affiliations:** Keck School of Medicine, University of Southern California, Los Angeles, CA 90033, USA; afyang@usc.edu (A.F.Y.); leeemily@usc.edu (E.L.); mahsa.babaei@med.usc.edu (M.B.)

**Keywords:** methadone, orthopedic surgery, postoperative pain, analgesia, scoping review

## Abstract

Background/Objective: The present study is a scoping review of the literature from Medline, PubMed, and Scopus databases from 2000 to 2025. Studies were selected based on predefined criteria, including the intraoperative administration of methadone during orthopedic surgery and the reporting of outcomes such as pain scores, opioid consumption, or adverse effects. A qualitative synthesis of the findings from 13 selected studies was performed to evaluate the existing literature on the efficacy and safety of perioperative methadone for postoperative pain management in orthopedic surgery. Methods: Studies were selected based on predefined criteria, including the intraoperative administration of methadone during orthopedic surgery and the reporting of outcomes such as pain scores, opioid consumption, or adverse effects. A qualitative synthesis of the findings from 13 selected studies was performed. Results: Randomized controlled trials in adult spine surgery consistently showed that intraoperative methadone (0.2 mg/kg) significantly reduced postoperative opioid consumption by up to 50% and lowered pain scores for up to 72 h compared to other opioids. Similar benefits were observed in pediatric spine surgery, particularly with multimodal regimens including methadone, which reduced total opioid use by as much as 76%. However, for arthroscopic knee surgery, morphine provided superior analgesia compared to methadone. The safety profile of perioperative methadone appears acceptable, with no major adverse events reported when dosed appropriately. Conclusions: Perioperative methadone is an effective analgesic adjunct that reduces postoperative pain and opioid requirements, especially in major spine surgery when integrated into a multimodal strategy. Its efficacy varies by surgical context, and further high-quality research is needed to define optimal dosing and its role in other orthopedic procedures.

## 1. Introduction

Chronic Post-surgical Pain, or CPSP, is a pain syndrome lasting for two or more months that develops after both major surgeries and minor procedures [1]. Acute pain in the immediate post-operative period has been well-described as a major risk factor to developing CPSP [2,3,4,5]. In one recent study, Fletcher et al. revealed that for every 10% increase in percentage of time in severe pain, there was an associated 30% increase in CPSP incidence at the 12-month mark post-operatively. The same study found that among 3120 patients undergoing surgery in a European registry, the incidence of moderate to severe CPSP after 12 months post-operatively was 11.8% (95% CI 9.7 to 13.9) [6]. Thus, utilizing regional anesthesia techniques and aggressive intraoperative multimodal analgesia is of paramount importance in preventing CPSP among patients [7].

One population that is highly susceptible to CPSP is orthopedic surgery patients [6,8]. One prospective cross-sectional study found that among general surgical patients at a level 1 trauma center, the highest incidence rates of CPSP after 2 years were orthopedic/trauma patients (57%), followed by general (28%) and vascular (15%) surgeries [8]. Rates of CPSP for orthopedic surgery patients range as high as 42% for total knee arthroplasties 3 months post-operatively to 15% for pediatric spinal fusions 5 year post-operatively [9,10]. Interestingly, one meta-analysis noted that in their orthopedic surgery subgroup analysis, pre-operative pain was a more important risk factor for CPSP than younger age and female sex [11].

While post-operative opioid analgesia remains a crucial component to minimizing the risk of CPSP in orthopedic surgery patients, opioid-prescribing patterns in orthopedic surgery are a large contributor to the opioid epidemic [12]. Thus, the use of intraoperative methadone, a powerful N-methyl-D-aspartate receptor antagonist, is seeing growing popularity due to its long elimination half-life (24–36 h), stable blood concentrations after a single dose, and high oral bioavailability [13]. These prolonged analgesic effects were evident in one systematic review and meta-analysis that revealed that intraoperative methadone use reduced postoperative pain scores up to 48 h after surgery as well as postoperative opioid consumption up to 72 h after cardiac, major spine, and abdominal surgeries compared to other opioids, including morphine, fentanyl, sufentanil, or remifentanil [14]. A recent scoping review revealed that perioperative methadone use during spinal surgeries was associated with significantly lower postoperative pain scores and perioperative opioid consumption [15]. Whether methadone provides the same beneficial reductions in post-operative opioid consumption and pain scores across all orthopedic surgeries remains unclear.

## 2. Materials and Methods

A thorough literature search was performed using Medline, PubMed, and Scopus from January 2000 to December 2024, with the last search performed on 14 February 2025. To broaden the scope, Google Scholar was also reviewed; however, it served mainly for identifying additional references rather than for structure screening. The search strategy incorporated the keywords “methadone use” and “orthopedic surgery” to capture relevant studies. Complete search terms with last search date are available in the Supplementary Files.

Initial screening of study title and abstracts was performed on the platform Covidence, which automatically removed duplicate studies. Two reviewers performed full text screening and only studies that both reviewers agreed upon were included in the analysis. Studies were included if they involved human participants who received methadone intraoperatively during orthopedic surgery and reported on at least one of the following outcomes: pain scores, postoperative opioid use, time to first opioid administration, respiratory depression, or other side effects. Studies were excluded if they were case reports, interviews, literature reviews, non-English studies, animal studies, and articles published before 2000.

From each included study, relevant information was extracted, including study design, patient population, surgical context (inpatient vs. outpatient), methadone dosing, and reported outcomes. A qualitative synthesis was then conducted to summarize trends related to the use of methadone in orthopedic procedures.

## 3. Results

**Figure 1:**
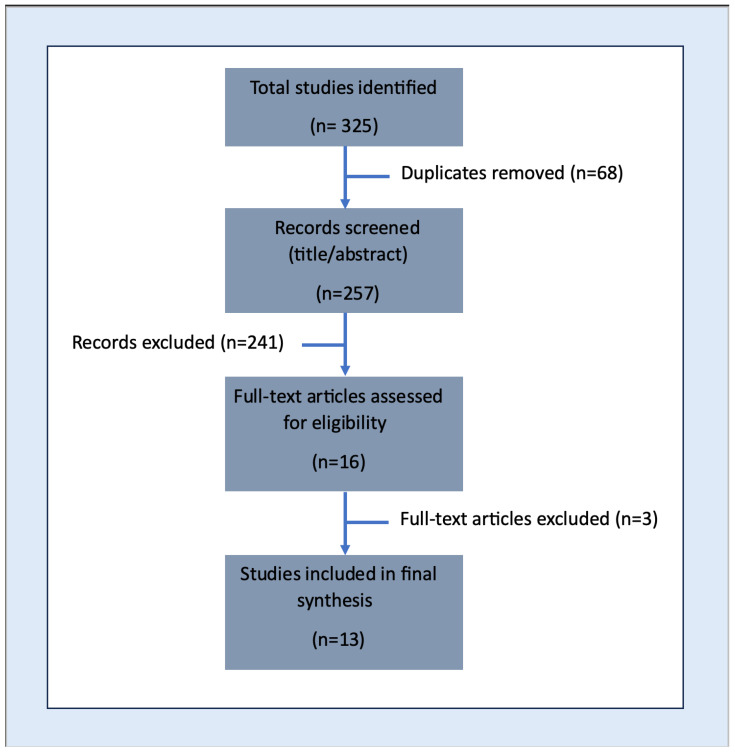
This is a PRISMA Diagram which depicts the selection of articles for this scoping review.

The initial search yielded 325 articles. After removing 68 duplicates, 257 titles and abstracts were screened. Studies that did not meet inclusion criteria were excluded resulting in 16 full-text articles for detailed review. Of these, 13 studies met all inclusion criteria and were included in the final analysis (Figure 1).

A summary of the included studies is provided in Table 1, which outlines study design, population characteristics, methadone dosing regimens, comparison groups, outcomes, and main findings (Table 1).

### 3.1. Randomized Controlled Trials

In adult spine surgery, RCTs consistently show that intraoperative methadone reduces postoperative pain and opioid usage. Gottschalk et al. (2011) randomized 29 adults undergoing multilevel thoracolumbar spine surgery with instrumentation and fusion to either single-dose methadone (0.2 mg/kg) prior to surgical incision or continuous sufentanil infusion of 0.25 μg/kg/h after a loading dose of 0.75 μg/kg [16]. Methadone halved opioid use at 48 (median [inter-quartile range] Morphine Milligram Equivalent (MME): 25 [16.5/31.5] mg vs. 63 [27.3/86.1] mg, *p* = 0.023) and 72 h (MME: 15 [8.8/27.8] mg vs. 34 [19.9/91.5] mg, *p* = 0.024). Pain scores were similarly reduced by half at 48 h (2.8 vs. 4.8, *p* = 0.026). No significant differences were found in side effect rates. Similarly, Murphy et al. (2020) found that 0.2 mg/kg methadone at anesthesia induction in 120 adults undergoing posterior fusion compared to 2 mg hydromorphone at surgical closure significantly reduced postoperative hydromorphone use on POD 1–3 (median 4.6 vs. 9.9 mg on POD1, *p* < 0.001) and lowered pain scores at most time points [17]. In the same study, Murphy et al. also found benefits of methadone usage in cardiac surgery; however, those results fall outside of this paper’s scope of orthopedic surgery. In pediatric spine surgery, a blinded RCT by Martin et al. (2018) compared remifentanil alone to remifentanil combined with either methadone (0.1 mg/kg) IV over 15 min or magnesium in adolescents having posterior fusion [18]. Total inpatient opioid (hydromorphone) use was significantly lower with methadone (0.26 ± 0.10 vs. 0.34 ± 0.11 mg/kg, *p* = 0.035), although pain scores did not differ. No significant differences were seen between the remifentanil + magnesium group and the remifentanil group.

In adult knee surgery, RCTs demonstrate that intraoperative methadone is less effective than morphine for postoperative pain. Arti et al. (2011) randomized 150 candidates for knee arthroscopic ACL reconstruction to postoperative analgesia with intra-articular bupivacaine and epinephrine with one of either 5 mg methadone, 5 mg morphine, 37.5 mg pethidine, 100 mg tramadol, or 0.5 mL normal saline [19]. Though methadone and morphine lead to similar amounts of postoperative opioid usage in the third 4 h after surgery (3.6 ± 0.56 vs. 2.4 ± 8.6), morphine significantly decreased pain scores relative to methadone (2.4 ± 0.8 vs. 5.2 ± 1.4). Arti et al. (2013) later randomized 140 patients who received arthroscopic meniscectomies to postoperative analgesia with bupivacaine and epinephrine with one of either 5 mg methadone, 5 mg morphine, 50 mg meperidine, or 5 mL normal saline [20]. The morphine group reported significantly decreased pain (2.02 ± 3.4 vs. 3.87 ± 3.3) and requested half the amount of analgesia compared to the methadone group (10.5 ± 9.4 vs. 22 ± 15.3 mg).

### 3.2. Non-Randomized Studies

#### 3.2.1. Pediatric Spine (Prospective Cohorts with Historical Controls)

Several prospective pediatric studies report similar benefits in decreased opioid usage with perioperative methadone. In a historically controlled trial of adolescent idiopathic scoliosis surgery, Tams et al. (2020) compared a retrospective control group with a new clinical pathway incorporating intraoperative methadone (0.25–0.4 mg/kg), gabapentin and acetaminophen multimodal analgesia [21]. The methadone-based pathway significantly decreased 24 h pain scores (mean [95% confidence interval]: 4.8 [4–6] to 3.4 [2–4], *p* = 0.03), reduced total 24 h opioid use by 76% (41 [29–51] mg to 10 [4–17] mg, *p* < 0.001), and shortened hospital stay by one day (4 [3–6] days to 3 [3–5] days, *p* = 0.001). Ye et al. (2020) similarly implemented a scheduled postoperative methadone regimen for posterior instrumented spinal fusion in 61 adolescent idiopathic scoliosis patients versus 61 controls who received intrathecal morphine and other adjuncts in a retrospective matched cohort [22]. The methadone-based multimodal group had significantly shorter length of stay (median 2 vs. 3 days, *p* < 0.001), lower total inpatient morphine consumption (mean 98 mg vs. 128 mg, *p* < 0.001), and faster return of bowel function (1 day vs. 2 days; *p* < 0.001). Pain scores were initially higher on POD0 with methadone (reflecting minimal immediate anesthesia), but became similar on POD1, and were lower by POD2 in the methadone group. Mean opioid usage mirrored this trend and ultimately resulted in decreased total opioid usage. Sadhasivam et al. (2021) administered multiple small doses of methadone (0.1 mg/kg intraop then q12h × 3–5 doses) in a multimodal regimen for PSF and pectus excavatum repair [23]. They reported minimal daily opioid requirements (median 0.66 mg/kg morphine equivalents) and no respiratory depression or QT prolongation in 38 children. These results suggest that a methadone-based multimodal approach can safely enhance patient analgesia and reduce overall opioid use in pediatric spine patients with minimal adverse side effects.

#### 3.2.2. Pediatric Spine (Retrospective Cohorts)

Other single-institution cohorts have retrospectively examined methadone in the setting of existing protocols. Mok et al. (2022) compared three analgesic regimens following pediatric PSF: standard PCA opioids (*n* = 26), PCA with pre-incisional methadone (*n* = 39), and pre-incisional methadone with scheduled methadone without PCA (*n* = 22) [24]. The group receiving intraoperative plus scheduled methadone (no PCA) used significantly less opioids overall than PCA-only or PCA with methadone groups (mean 0.18 vs. 0.33 and 0.30 mg/kg hydromorphone equivalents, *p* < 0.001). There were no significant differences in pain scores, sedation levels, or length of stay. Shaw et al. (2021) analyzed 26 AIS patients given single-dose methadone within an ERAS pathway versus 52 matched controls (no methadone) [25]. They found no significant difference in total opioid use or pain scores between groups during hospitalization. Kirk et al. (2024) retrospectively showed that of 111 adolescents undergoing PSF for AIS, those receiving intraoperative IV methadone were more likely to be discharged on POD1 (36% discharged POD1, *p* = 0.02) without higher rates of ED visits or hospital readmissions [26]. Thus, while most pediatric retrospective analyses report opioid reductions and shorter recovery with methadone pathways, some have found neutral effects when methadone was a single change.

#### 3.2.3. Other Surgical Settings

Chan et al. (2017) studied 36 chronic methadone maintenance patients undergoing total knee arthroplasty in a retrospective case–control study [27]. Those on chronic methadone used significantly more inpatient opioids (higher median daily use and higher PCA usage, *p* < 0.001) and experienced longer hospital stays compared to matched controls. In a larger prospective cohort of 240 adults, Petrosan et al. (2021) matched surgical patients who received intraoperative methadone to controls [28]. They found higher 24–72 h opioid consumption in the methadone group (mean 24 h MME 142.6 vs. 84.5, *p* = 0.003) with no difference in pain score; however, opioid-naive subgroup analyses demonstrated lower 48 h MME (*p* = 0.024) and lower pain scores (*p* = 0.037) in the methadone group. In summary, non-randomized evidence outside of spine and cardiac surgery is mixed. Many pediatric and spinal studies report opioid-sparing and faster recovery with methadone regimens, whereas some broader studies observed no benefit or even increased early opioid use with methadone unless patients were opioid-naive.

#### 3.2.4. Stratification Summary

In summary, intraoperative methadone’s analgesic efficacy varies by surgical type, age group, and dosing regimen. Patients undergoing spine surgery exhibit the strongest and most robust benefits of intraoperative methadone, with both adult and pediatric patients demonstrating reductions in opioid use and pain scores. In contrast, studies of non-spine procedures such as arthroscopic knee surgery demonstrated decreased benefit compared to morphine. With adult patients, a single intraoperative methadone bolus was sufficient to achieve analgesic benefit; in pediatric patients, repeated small-dose regimens appeared to be more effective and were associated with an acceptable safety profile. Tailoring methadone use to patient subgroup and surgical context is critical to optimizing potential analgesic benefit.

## 4. Discussion

### 4.1. Analgesic Efficacy

The studies discussed in this review generally support the perioperative analgesic efficacy of methadone, especially in spinal surgery. Randomized trials in adults have consistently shown that a single intraoperative dose of methadone of typically 0.2 mg/kg can reduce opioid requirements by approximately 50% and lower postoperative pain scores through the first 72 h postoperatively [16,17]. Retrospective studies in children having spinal surgery have also demonstrated similar analgesic benefits; where scheduled, small-dose methadone regimens as part of multimodal analgesic plans lead to substantial reductions in overall opioid use (up to 76%) and improved postoperative pain control [21,22]. These findings are consistent with other systematic reviews regarding the analgesic efficacy of methadone in spine surgery [29].

However, the efficacy of methadone appears to vary by surgery type. A recent study by Whitaker et al. demonstrated significant benefits of preoperative methadone compared to preoperative oxycodone in both postoperative pain and opioid usage [30]. In contrast, two RCTs studying intra-articular methadone for arthroscopic knee surgeries found that while methadone did modestly reduce postoperative analgesic needs, morphine consistently produced superior analgesia [19,20].

Methadone’s decreased efficacy in knee surgeries may be explained by both pharmacologic and procedural effects. Intra-articular morphine provides highly localized pain relief via peripheral effects on synovial opioid receptors [31,32]; methadone’s primary advantages including long half-life and NMDA receptor antagonism may be more relevant in systemic administration as in spine surgery [13,33]. Additionally, knee arthroscopy is a relatively short, lower-pain procedure in which methadone’s prolonged analgesic profile may offer little additional benefit compared to morphine. These mechanisms may explain why methadone does not perform as well as morphine in knee surgery RCTs.

Furthermore, methadone’s role in analgesia may vary with its use in ERAS protocols and multimodal analgesic pathways. Some studies such as Shaw et al. failed to demonstrate a benefit when methadone was added to existing optimized ERAS pathways, suggesting that its effect may be limited in highly structured perioperative protocols [25]. Conversely, when incorporated other protocols as in the historical-controlled studies by Tams et al. and Ye et al., methadone contributed to notable reductions in pain and opioid use [21,22]. These findings demonstrate that methadone may be a helpful adjunct within ERAS protocols for select orthopedic populations, but its benefit may depend on the specific perioperative pathway.

Overall, these findings underscore methadone’s role as a potent intraoperative analgesic, especially in high-pain surgeries when integrated into multimodal strategies. However, its analgesic advantage is not universal and appears to be less pronounced or even absent in lower-pain or localized surgical contexts, such as knee arthroscopy.

### 4.2. Safety Profile

A common concern regarding analgesic use of methadone is the risk of adverse side effects such as respiratory depression [29,34]. In the pediatric multidose study by Sadhasivam et al., repeated small doses of methadone were not associated with any episodes of respiratory depression or QT prolongation [23]. Adverse effects such as nausea or vomiting were significantly correlated with MME/kg rather than the methadone dose itself [23]. In chronic users, however, maintenance methadone patients clearly had higher analgesic requirements, reflecting opioid tolerance rather than perioperative risk [27]. Thus, with careful dosing and monitoring, methadone’s safety profile appears acceptable with minimal severe adverse effects. No study reported fatal events or unmanageable side effects directly attributable to methadone when used perioperatively.

### 4.3. Clinical Impact

By lowering pain and opioid needs, methadone can accelerate patient discharge. Several studies found shorter hospital stays, decreased pain management referral usage, and faster return of bowel function when methadone was used [21,22,26]. These benefits may vary by institution, as some studies showed no added reduction in the length of stay with methadone.

The evidence on functional outcomes beyond immediate recovery is limited. No study has conclusively shown that methadone reduces long-term CPSP or opioid dependence. However, 3-month follow-up of one RCT in patients undergoing complex spine surgery revealed a significantly lower proportion reporting any CPSP (50% vs. 85%, *p* = 0.010) and any analgesic requirement (10% vs. 41%, *p* = 0.009) compared with controls [17]. This suggests that methadone can prevent the development of CPSP following high-pain orthopedic procedures, with similar benefits observed in cardiac surgery patients supporting this evidence. One possible explanation for this is methadone’s NMDA receptor antagonism, which has been found to decrease central sensitization, a key mechanism of CPSP development [35,36]. Notably, however, the protective effects diminished by six to twelve months, indicating that methadone may only delay rather than prevent CPSP.

These findings underscore the potential for methadone to reduce both immediate postoperative opioid requirements and improve long-term recovery. Further investigation in other populations is warranted to understand whether these benefits extend more generally in CPSP. Overall, methadone appears most effective in lowering opioid burdens, improving analgesia quality, and enhancing recovery, but its long-term benefits remain to be determined.

### 4.4. Limitations and Future Directions

The current literature has notable gaps. Many studies are small, single-center, and non-randomized, which limits generalizability. Publication bias can also influence the existing literature base as positive findings are more likely to be published than neutral or negative results. Most studies to date have been conducted at single institutions, which can limit generalizability due to variation in local institutional protocols and patient demographics. Dosing regimens vary widely (single bolus vs. multiple doses, 0.1–0.4 mg/kg, IV vs. oral), making it difficult to define a single optimal strategy. Some studies focus only on pediatric populations or specific types of orthopedic surgeries, limiting the generalizability of findings to adult patients or broader surgical context. Adverse-event reporting is also inconsistent; most trials reported only common side effects, without systematic QT monitoring or follow-up of delayed respiratory events.

Both adults and children benefit from methadone, but the effects may differ by age. Adults reliably experienced opioid-sparing and pain relief with a single intraoperative dose. In adolescents, single doses helped modestly, but it appears multiple small doses are more effective and safer [23]. One possible rationale for repeated small-dose regimens in children is that developmental pharmacokinetics which can lead to differences in methadone metabolism and clearance may make single large boluses less predictable in pediatric patients [37]. Developmental differences in nociceptive pathways can influence pain physiology, requiring prolonged or repeated dosing to maintain adequate analgesia [38]. These factors could support the use of repeated small-dose regimens for methadone in pediatric surgical populations and highlight the need for further investigation of pharmacokinetic and pharmacodynamic studies in this age group.

Future research should address these uncertainties: large multicenter RCTs are needed to compare methadone (with defined dosing) against standard regimens across varied surgeries. Studies should stratify by opioid tolerance status, age group, and surgical risk. The interplay with other non-opioid analgesics including ketamine and regional blocks is promising but remains understudied. Finally, long-term outcomes such as chronic pain rates, postoperative opioid use, and functional recovery should be evaluated to clarify whether the early benefits of methadone translate into lasting improvements.

## 5. Conclusions

In summary, perioperative methadone appears to offer strong analgesic efficacy with an acceptable safety profile in complex surgeries, especially when embedded in a multimodal regimen. Incorporating methadone into perioperative care can reduce opioid exposure and enhance recovery without added harm. Nonetheless, due to heterogeneity of the evidence, clinicians should consider patient factors (age, comorbidities, and baseline opioid use) and institutional protocols when deciding on methadone use. Further rigorous trials are needed to refine its role within specific surgical settings.

## Figures and Tables

**Table 1:** Summary of studies evaluating intraoperative methadone use in orthopedic surgery.

Study First Author (Year)	Study Design	Population (N)	Surgical Context	Methadone Dose/Timing	Comparison Group(s)	Outcomes Reported	Main Findings
Gottschalk (2011) [16]	RCT	29 adults	Multilevel thoracolumbar spine fusion	0.2 mg/kg IV before incision	Continuous sufentanil infusion (0.25 μg/kg/h after loading dose)	Opioid use, pain scores, adverse effects	Methadone ↓ opioid use ~50% at 48–72 h; ↓ pain scores; no ↑ adverse effects
Murphy (2017) [17]	RCT	120 adults	Posterior spinal fusion	0.2 mg/kg IV at induction	2 mg IV hydromorphone at closure	Opioid use, pain scores	Methadone ↓ opioid use POD1–3; ↓ pain scores
Martin (2018) [18]	RCT	60 adolescents	Posterior spinal fusion	0.1 mg/kg IV over 15 min (with remifentanil)	Remifentanil alone or remifentanil + magnesium	Opioid use, pain scores	Methadone group ↓ total opioid use vs. controls; pain scores NS
Arti (2011) [19]	RCT	150 adults	Arthroscopic ACL reconstruction	5 mg intra-articular methadone	5 mg morphine, 37.5 mg pethidine, 100 mg tramadol, saline	Opioid use, pain scores	Morphine provided superior analgesia vs. methadone; methadone not superior to tramadol/pethidine
Arti (2013) [20]	RCT	140 adults	Arthroscopic meniscectomy	5 mg intra-articular methadone	5 mg morphine, 50 mg meperidine, saline	Opioid use, pain scores	Morphine ↓ pain and analgesic requests vs. methadone; methadone less effective
Tams (2020) [21]	Historically controlled trial	60 adolescents	Posterior spinal fusion (AIS)	0.25–0.4 mg/kg IV intraop + multimodal (gabapentin, acetaminophen)	Historical controls without methadone pathway	Pain scores, opioid use, LOS	Methadone pathway ↓ pain scores, ↓ opioid use by 76%, ↓ LOS by 1 day
Ye (2020) [22]	Retrospective matched cohort	122 adolescents	Posterior spinal fusion (AIS)	Scheduled postop IV methadone (multimodal pathway)	Intrathecal morphine + adjuncts	Opioid use, LOS, bowel function, pain scores	Methadone group ↓ opioid use, ↓ LOS, faster bowel recovery
Sadhasivam (2021) [23]	Prospective single-arm study	38 children	Posterior spinal fusion and pectus excavatum	0.1 mg/kg intraop then q12h × 3–5 doses	No comparator (single-arm study within multimodal regimen)	Opioid use, safety (respiratory, QT)	Very low opioid requirements; no respiratory depression or QT issues
Mok (2022) [24]	Retrospective cohort	87 adolescents	Posterior spinal fusion	Pre-incisional methadone + scheduled postop vs. PCA-based regimens	PCA opioids only; PCA + single-dose methadone	Opioid use, pain scores, LOS	Scheduled methadone group used significantly less opioids; pain/LOS NS
Shaw (2021) [25]	Retrospective matched cohort	78 adolescents	Posterior spinal fusion (ERAS pathway)	Single intraop IV methadone	ERAS without methadone	Opioid use, pain scores	No significant difference in opioid use or pain
Kirk (2024) [26]	Retrospective cohort	111 adolescents	Posterior spinal fusion (AIS)	Single intraop IV methadone	No methadone	LOS, ED visits, readmissions	Methadone group more likely to discharge POD1; no ↑ readmissions or ED visits
Chan (2017) [27]	Retrospective case–control	36 adults	TKA in methadone-maintenance patients	Chronic methadone users vs. opioid-naïve controls	Opioid-naïve TKA patients	Opioid use, LOS	Methadone-maintenance pts required more opioids, longer LOS
Petrosan (2021) [28]	Prospective matched cohort	240 adults	Mixed surgeries (orthopedic subgroup included)	Intraop IV methadone	Matched controls (no methadone)	Opioid use, pain scores	Overall ↑ opioid use with methadone; in opioid-naïve subgroup, methadone ↓ opioid use and pain

↓ indicates decrease, ↑ indicates increase.

## Data Availability

No new data were created or analyzed in this study. Data sharing is not applicable to this article.

## Supplementary Materials

The following supporting information can be downloaded at: https://www.mdpi.com/article/10.3390/healthcare13192431/s1.
