# Supplementary material for: Perioperative Methadone in Orthopedic Surgery: A Scoping Review
Source: Healthcare (Basel). 2025 Sep 25;13(19):2431. doi: 10.3390/healthcare13192431 (PMC12524262; doi:10.3390/healthcare13192431)
Supplement: Supplementary file 1 [file healthcare-13-02431-s001.zip › healthcare-3844699-supplementary.pdf]

Search terms: “orthopedic surgery” and “methadone use” – exact search query: orthopedic surgery methadone use

Last search date: Feb 14, 2025
